# Food Is Always Close By: An Assessment of the Healthiness of the Adolescent Home and School Food Environments in Northern Vietnam

**DOI:** 10.1016/j.cdnut.2026.109402

**Published:** 2026-06-17

**Authors:** Gabriela Fretes, Nicholus T Zaw, Phuong H Nguyen, Agnes Le Port, Kim Maasen, Elise F Talsma, Mai T Truong, Trang TT Tran, Inge D Brouwer, Marie T Ruel, Jef L Leroy

**Affiliations:** 1 Nutrition, Diets, and Health Unit, International Food Policy Research Institute, Washington, DC, United States; 2 Montpellier Interdisciplinary Centre on Sustainable Agri-Food Systems (UMR MoISA), French National Research Institute for Sustainable Development, Montpellier, France; 3 Division of Human Nutrition and Health, Wageningen University & Research, Wageningen, The Netherlands; 4 National Institute of Nutrition, Hanoi, Viet Nam; 5 CGIAR System Organization, Nairobi, Kenya

**Keywords:** food environments, adolescents, spatial analysis, urbanicity, Vietnam

## Abstract

**Background:**

Adolescents are vulnerable to rapidly evolving food environments. Urbanization is widely thought to contribute to less healthy food environments, and school food environments are often considered less healthy than those at home. These associations, however, have not been quantified.

**Objectives:**

Assess differences in adolescent food environments in Northern Vietnam by setting (home compared with school) and area (rural, peri-urban or urban).

**Methods:**

We collected cross-sectional data on adolescents (*n* = 3005) and surveyed all outlets (*n* = 6194) that sold food or beverages around adolescents’ homes and schools (*n* = 13) in 3 sites that differed in urbanicity. We calculated distances from home and school to the nearest outlet selling select food groups and measured outlet density and diversity of foods offered (within a 100-m radius). We analyzed differences by urbanicity and by home/school environment using regression models, accounting for clustering at the commune level.

**Results:**

Both unhealthy food groups (UFG) (e.g., sugar-sweetened beverages, sweets) and healthy food groups (HFG) (e.g., fruits, nuts/seeds) were closer to adolescents’ homes in the urban (∼21‒50 m) than the peri-urban (∼75‒200 m) and rural (∼155‒270 m) area. Fruits and vegetables were consistently further away than UFG across all areas in home environments, although still close by (∼ 50‒270 m). Urban adolescents were exposed to the greatest density and diversity of both HFG and UFG close to home and school. There were no differences in healthiness of home and school food environments.

**Conclusions:**

Unhealthy foods were more accessible (in distance) and available (in density) than fruits and vegetables across all settings, but differences were small, and food was always close by. Diversity of HFG and UFG was the greatest in urban areas. Healthy foods should be promoted and made the preferred choice through nudging interventions at point-of-purchase, combined with restrictions on the marketing and availability of unhealthy foods.

## Introduction

Food environments—the places where individuals interact with the broader food system to acquire and consume food [1]—are undergoing rapid and unprecedented transformation. These shifts are driven by urbanization, economic growth, and evolving consumer needs and lifestyles, especially in low- and middle-income countries (LMICs) [2]. As a result, consumers are rapidly shifting from traditional diets rich in minimally processed foods such as legumes, vegetables, and grains to diets containing a higher proportion of animal source foods and ultraprocessed foods (UPF) [3,4]. Because dietary shifts are shaped by the types of foods available and accessible to consumers, understanding food environments is essential to support healthy dietary choices.

Recent reviews on food environments note that most research to date has focused on high-income countries [5,6], whereas evidence from LMIC is only beginning to emerge [5,7,8]. A systematic review on urban food environments in LMICs found that greater availability (presence, density, or number of) and accessibility (proximity) of outlets selling fruits and vegetables was associated with higher intakes of fruits and vegetables [7]. A food environment scoping review focused primarily on African LMICs concluded that urbanization was associated with greater and more stable access to a wider diversity of foods, including both nutritious options and UPFs and unhealthy convenience foods. This pattern likely reflects a shift toward the consumption of less healthy diets, contributing to increased risks of overweight, obesity, and diet-related noncommunicable diseases [2].

Adolescents are particularly vulnerable to the effects of rapidly changing food environments. With increased pocket money (i.e., money that adolescents are given as an allowance) and mobility, they have greater autonomy to purchase and consume foods around their home or school [9,10]. Their food choices are shaped by the accessibility and affordability of foods around them, as well as peer influence and pervasive food marketing [[9], [10], [11]]. School food environments are particularly important because adolescents spend much of their day at school. Adolescents have been reported to perceive the home food environment as healthier than the school food environment [12,13]. The availability of healthy foods in and around schools can promote healthier dietary habits [14], whereas easy access to prepared foods and UPFs may encourage less healthy diets [9,[15], [16], [17]]. Despite growing recognition of these issues, research on adolescents’ food environments in LMICs remains scarce [18]. Vietnam’s rapid economic growth and urban expansion make it a unique setting for studying food environments. Prior work in Vietnam showed a strong rural-urban gradient in proximity to, and density of food outlets, with urban dwellers being consistently closer to outlets and the density of outlets being 3 to 4 times higher in urban, compared with peri-urban and rural areas [19]. The study, however, was conducted in adults and did not compare the availability or accessibility of healthy foods compared with unhealthy foods or differences between home and school food environments.

The aims of this study were to assess the healthiness of the food environment among adolescents in Northern Vietnam and to explore potential differences by urbanicity (rural, peri-urban, urban area) and between home and school food environments. We hypothesized that availability of and accessibility to unhealthy foods among adolescents from low- and middle-income neighborhoods would decline along the urban-peri-urban-rural continuum. It was also hypothesized that school food environments would be less healthy than home food environments, with higher availability of unhealthy foods that could be easily accessed around schools in low- and middle-income neighborhoods.

## Methods

### Study design, setting, and sampling

Data were collected using cross-sectional surveys undertaken in low- and middle-income communes in 3 areas in Northern Vietnam: a rural district (Moc Chau in Son La province) and a peri-urban (Dong Anh in Hanoi) and urban district (Dong Da, an urban district also in Hanoi).

#### Selection of communes

Within each study district, we ranked all communes based on a wealth and education index using the most recent 2019 Census administrative data. Communes are the lowest administrative unit in the 3-tiered local government system, which consists of *1*) province/city, *2*) district/town, and *3*) commune/ward. We selected lower-income communes where both a lower (grades 6‒9) and a higher (grades 10‒12) secondary public school were present. To reach the required sample size of adolescents for this study, 3 communes in Moc Chau, 2 communes in Dong Anh, and 2 communes in Dong Da were needed. We selected the communes with lower index rankings among the eligible communes. Additional details of the sampling strategy are described elsewhere [20].

#### Sample size

For the sample size estimation, we considered a design effect of 2 to account for the 2-stage sampling design (i.e., selection of households within study clusters), a 20% allowance for potential missing data, a statistical power of 80%, and a 5% significance level. Based on these assumptions, a sample of 960 adolescents per site was deemed sufficient to detect a 17% point difference in the prevalence of any outcome between 2 groups of equal size, assuming a baseline prevalence of 50% in 1 group (the most conservative scenario). This corresponds to a Cohen’s *d* effect size of 0.35, which is generally considered a moderate effect size, falling between the conventional benchmarks for small (*d* = 0.20) and medium (*d* = 0.50) effects [21]. Thus, we aimed to survey 2880 adolescent households across the 3 study sites.

#### Study participants

Adolescents 11 to 19 y old residing in the same communes as their schools were eligible to participate in the study. Adolescents with a disability or a physical or mental health issue were ineligible, as these conditions could have made it difficult to participate in key aspects of the study, such as the dietary recall and the anthropometric assessment [20]. Sampling was conducted through schools using probability-proportional to size, with classes and students randomly selected to ensure age and sex (i.e., biological sex determined at birth) balance. Sampling procedures were adapted to achieve the target sample size in Dong Anh and Dong Da. In Dong Anh, there was a shortage of students in higher secondary grades, and instead of selecting 3 classes per grade (as planned), all classes were included to meet the sample size. In Dong Da, this strategy was not enough, so we included adolescents living outside of the commune where the school was located but still within the district of their schools. Further details on the sampling are available elsewhere [20].

#### Food outlets

We surveyed all outlets that sold any food or beverage in each commune. Considering the recruitment of adolescents outside the originally sampled communes in Dong Da, the food outlet survey was expanded. The study resources allowed us to survey food outlets in an additional 3 communes, which were chosen to maximize the number of adolescents for whom the food environment information would be available.

### Data collection

Household and adolescent data were collected through home visits between December 2022 and April 2023. The survey included information on adolescents’ age, sex, and education, maternal education, household global positioning system (GPS) coordinates, household head sex and education, household assets, access to services (drinking water, sanitation, and cooking fuel), and household food insecurity. Household food insecurity was calculated using the Household Food Insecurity Access Scale [22]. Sex refers to biological sex determined at birth and was recorded in the household roster [What is (NAME’s) sex? 1 male 2 female].

The food outlet survey was conducted from February 2023 to March 2023. Maps with streets delimited were used to guide enumerator teams and for field monitoring to check for any areas and streets that were not covered within the commune administrative boundaries. Data collected included food outlet GPS coordinates, outlet typology, availability of different foods sold (i.e., single foods and meals), food prices, and marketing and promotion. Food prices and marketing and promotion results will be presented in a different paper. Data were collected through direct observation by trained enumerators. In cases where observation alone was insufficient, enumerators interacted with outlet owners or employees. If an outlet was closed at the time of the survey, enumerators revisited the areas with closed outlets on a different day.

Considering the diversity of food offerings in Vietnam, it was not feasible to collect data on all foods offered in the surveyed outlets. Therefore, we limited data collection to foods included in the food list from the dietary quality questionnaire, which comprises 29 food groups. The Vietnam-adapted (and validated) dietary quality questionnaire has 7 foods in each food group, which captures 98% of what people eat in Vietnam [23]. We added a few key foods that were of interest for the study, including different types of milk, biscuits, bubble milk tea, and frozen foods, among others. For meals/dishes in restaurants, we constructed a pre-specified meals/dish list based on previous research on food consumed away from home [24]. The meals/dishes list included information on the cooking method (e.g., stir-fried, fried) and, in most cases, the main ingredients of the dish (e.g., seafood and vegetables, stir-fried; fish, fried). At the end of the survey, enumerators took a picture of each outlet for supervision and outlet identification purposes.

Lastly, we collected GPS coordinates from the lower and upper secondary schools (*n* = 13) that adolescents attended. Prior to the start of data collection, all assessment tools were piloted in communes that were not part of the study to assess the feasibility of the tools. All data were recorded on tablet computers with integrated GPS using computer-assisted personal interviews technology via SurveyCTO software.

### Food categorization

We classified foods using the food groups in the global dietary quality score (GDQS) tool [25]. In the case of meals/dishes, we obtained the recipes, and each ingredient was classified into its respective GDQS food group. The GDQS provides a simple, standardized population-based measure of the healthiness of individual dietary intake. It comprises 25 foods groups: 16 healthy food groups (HFG), 7 unhealthy food groups (UFG) and 2 food groups considered unhealthy in excessive amounts (i.e., if consumed above a specified intake level) (UXFG) (Supplementary Table 1).

In addition to HFG, UFG, and UXFG, we also categorized foods by level of processing using the NOVA classification system for UPF. UPF are industrial formulations made mostly or entirely from substances derived from foods and additives. UPF are typically energy-dense; high in added sugars, sodium, and/or unhealthy fats, and have little to no whole ingredients in their composition [26]. Examples of UPF are flavored dairy drinks, packaged snacks, chips, chocolate, and processed meats. For sugar-sweetened beverages (SSBs), we used the WHO definition: “all types of beverages containing free sugars, and these include carbonated or non-carbonated soft drinks, fruit/vegetable juices and drinks, liquid and powder concentrates, flavored water, energy and sports drinks, ready-to-drink tea, ready-to-drink coffee and flavored milk drinks” [27]. Lastly, we also categorized fruits and vegetables. For fruits, we combined the following GDQS food groups: citrus fruits, deep orange fruits, and other fruits. For vegetables, we combined dark green leafy vegetables, cruciferous vegetables, deep orange vegetables, and other vegetables.

### Food environment measures

In this study, the food environment was defined as consisting of all food outlets in an adolescent home neighborhood and around their school. Our measurement approach was informed by Turner’s framework [1], which conceptualizes food environments as comprising multiple external (e.g., availability, vendor, and product properties) and personal (e.g., accessibility, convenience) dimensions. Although this framework provides a valuable conceptual structure, translating its dimensions into measurable constructs remains challenging, as there is limited consensus on how broad concepts such as accessibility or availability should be operationalized into specific, validated indicators. Moreover, it remains unclear which dimensions ultimately drive consumption decisions. Therefore, we assessed several food environment characteristics that capture different aspects of adolescents’ potential exposure to foods.

First, we described the types of outlets available at each study site. The outlet categories were created in consultation with local experts and refined through pre-testing to ensure contextual relevance (Supplementary Table 2). To assess proximity to outlets selling select food groups, we measured the straight-line (Euclidean) distance in meters from each adolescent’s home and school to the nearest food outlet selling select food groups (e.g., UPF, SSB, fruits and vegetables, among others) using geospatial coordinates collected via GPS. Distances were calculated using only outlets within the study area. No special consideration was taken for households and schools located near the border of the study area for the proximity measure.

We calculated the density of outlets, i.e., the total number of outlets per square kilometer and the number of outlets selling select food groups per square kilometer. Density measures were limited to a 100-m radius from each adolescent home and school, as we considered outlets falling within this radius to be easily accessible and thus an important part of the adolescent food environment. The density measures thus reflect the intensity of potential exposure to foods within adolescents’ immediate environments. For households and schools located near commune borders, portions of the 100-m radius around them extended beyond the study area, where outlet data were unavailable. To ensure comparability between border-adjacent locations and those located in the interior of communes, we excluded the area that fell outside the study boundary from the denominator. Density calculations were therefore normalized using only the area within the study site, rather than assuming a full 100-m radius for all observations.

The diversity of HFG, UFG, and UXFG was measured by counting unique food groups present within a 100-m radius around adolescents’ homes and schools. Each food group was counted only once, even if it appeared multiple times across outlets. For example, if 5 outlets within the 100-m radius each sold HFG items, the diversity score would count HFG once (presence/absence across outlets), not 5 times (frequency across outlets). This principle also applied to individual ingredients in restaurant meals/dishes. We did not consider the variety of items within each group. Higher scores indicated greater exposure to a variety of healthy or unhealthy food options.

We used MyGeodata cloud to create geospatial shapefiles for each location [28]. As the publicly available shapefiles for the study area were outdated, several corrections had to be made. We first corrected inaccuracies in commune boundary shapefiles by cross-checking administrative maps against Google Maps imagery and validating discrepancies with the field team. These corrections included resolving topological errors such as merging Moc Chau’s (rural) 2 polygons into a single shape and adjusting O Cho Dua’s (urban) extent to reflect its actual administrative coverage.

### Statistical analysis

Descriptive statistics (mean, SD, median, IQR, and frequencies) were calculated by geographical location and environment and are provided for all outcomes and additional variables (e.g., household and adolescent socio-demographic characteristics such as adolescent age, maternal education, and household assets) to describe the sample.

Distance measurements were winsorized at the 95th percentile to mitigate the influence of extreme values while preserving the distribution of the data. Most extreme values were present in the rural area (*n* = 131) where households were widely dispersed, whereas most food outlets were concentrated along the main road. Given the skewed distribution of distance data, mixed-effects quantile regression models were fitted at the 50th percentile to compare median distance across geographic locations and between home and school food environments. Shapiro-Wilk tests rejected normality for the distance measures (*P* < 0.001), supporting the use of quantile regression for median-based estimates (results not shown). Analyses assessing differences between home and school food environments were conducted at the adolescent level.

Due to the high frequency of 0 in the density data, mixed-effects Poisson regression models were used to assess differences in density across geographic locations [29]. Significant testing of differences across geographic locations was limited to the adolescent’s home food environment. Differences between home and school food environments were assessed using mixed-effects linear regression models with analyses conducted at the adolescent level.

Given the small number of schools (*n* = 13), no significant testing of differences in the school food environment by study site was conducted. All models included robust estimation of SEs adjusted for clustering at the commune level.

Area calculations and mapping of adolescents’ homes, schools, and food outlet geocoded locations were performed using the sf package in R software [30,31]. Outcomes were analyzed as continuous variables using Stata version 18 [32]. We used 2-tailed statistical tests, and statistical significance was assessed at 0.05 α level. As no simultaneous hypothesis testing was conducted, we did not correct for multiplicity [33].

### Sensitivity analysis

Kernel density estimation (KDE) was used as a sensitivity analysis to test whether results obtained using separate distance and 100-m density measures were robust to an alternative spatial exposure indicator that combines information on food group proximity and concentration without imposing hard thresholds. KDE is a method commonly used in spatial analysis to create a continuous surface (or “heatmap”) that highlights areas with higher or lower concentrations of points—in this case, food outlets. The method assigns more weight to outlets located nearby and less weight to those farther away, allowing us to capture not only how many outlets were present but also how densely they clustered around adolescents’ home or school environments.

KDE requires selecting a kernel function and a search radius (bandwidth), which defines how far from each outlet its influence extends. In this study, we used the Quartic (biweight) kernel, a standard method that gives progressively less influence to outlets further away. KDE surfaces were generated using QGIS software (version 3.36.1) with a 3-m pixel resolution to ensure detailed outputs. Finally, we extracted the density value at each adolescent’s home and school location from these KDE maps. Higher values represent greater concentrations of food outlets in the surrounding area.

In a second set of sensitivity analyses, we changed the 100-m radius to a 200-m and 400-m radius in the density and KDE analyses.

Lastly, we analyzed the analyses excluding households located <100 m from the commune boundaries (*n* = 258) to account for potential measurement errors due to households or schools being located near the boundary.

### Ethics

The study protocol was approved by the National Institute of Nutrition of Vietnam’s Ethics Committee (# 1573/QĐ-VDD) and by the International Food Policy Research Institute’s Institutional Review Board (PHND-22-1166). For the household and adolescent survey, written informed consent was obtained through parents’ or caregivers’ signatures, and written assent was obtained from adolescents. For the food outlet survey, the study’s objectives and procedures were explained to outlet owners or employees, and oral consent was obtained.

## Results

We surveyed 3005 adolescent households. For the home food environment analyses, households outside the areas where the food environment survey was conducted were excluded (*n* = 312). Among the 2693 households within the survey area, some had missing GPS coordinates (*n* = 30). Therefore, the final sample was 2663 adolescents. For the school environment analyses, 1 school in the peri-urban area was located outside the commune boundary and was excluded. The final sample was 12 schools (5 in the rural, 3 in the peri-urban, and 4 in the urban areas).

A total of 6194 food outlets were surveyed. Outlets that fell outside the commune boundary (*n* = 314) and those with missing GPS data (*n* = 38) were excluded, resulting in a sample of 5842 food outlets.

### Adolescent and household characteristics

Adolescents were on average 14 y old, 50% were female, and >90% lived with their mother (Table 1). Adolescents’ mode of transport to school varied across study sites, with using a motorbike (51%), using a bicycle (60%), and walking (58%) being most common in the rural, peri-urban, and urban areas, respectively. Households had on average 4 members. The level of education of the household head and the adolescent’s mother were higher in the urban area compared with the peri-urban and rural areas. More households in the urban and peri-urban areas owned assets such as digital television, computer/laptop, and microwave oven compared with rural households. The percentage of food-insecure households was significantly higher in the rural area (45%) compared with peri-urban and urban areas (<11%).

**TABLE 1:** Adolescent and household characteristics by geographical location

	Geographical location		
	Rural [a]	Peri-urban [b]	Urban [c]
	**Mean** ± **SD**	**Mean** ± **SD**	**Mean** ± **SD**
	*n* = 1067	*n* = 933	*n* = 663
**Adolescent**			
Age, years	14.0 ± 2.0	14.2 ± 2.0	13.7 ± 1.9
Female, %	50.8	49.5	50.8
Live with mother, %	92.0^[b]^	96.1^[a, c]^	91.9^[b]^
*Education, %*			
Lower secondary school	57.4	56.5	70.1
Higher secondary school	42.6	43.5	29.9
Mode of transportation to school, %			
Walking	12.9^[c]^	16.0^[c]^	58.4^[a, b]^
Bicycle	31.7^[b]^	59.9^[a, c]^	21.7^[b]^
Motorbike	51.3^[b, c]^	24.0^[a]^	19.5^[a]^
Bus/public transportation	3.8^[b, c]^	0.1^[a]^	0.5^[a]^
Car	0.3^[b, c]^	0.0^[a]^	0.0^[a]^
**Household**			
Size	4.5 ± 1.3	4.7 ± 1.2^[c]^	4.4 ± 1.2^[b]^
Head: male, %	69.3^[c]^	70.8^[c]^	49.5^[a, b]^
*Household head education, %*			
No/incomplete primary	1.5^[b, c]^	0.3^[a]^	0.2^[a]^
Primary level completed	37.8^[b, c]^	24.1^[a, c]^	3.1^[a, b]^
Lower secondary level completed	29.2^[b, c]^	24.9^[a, c]^	13.1^[a, b]^
Upper secondary level completed	18.2	22.4	25.9
Vocational level completed	5.5^[b, c]^	12.7^[a]^	12.6^[a]^
College or higher education completed	7.9^[b, c]^	15.5^[a, c]^	45.1^[a, b]^
*Maternal education, %*			
No/incomplete primary	2.0^[b, c]^	0.5^[a]^	0.0^[a]^
Primary level completed	36.5^[b, c]^	19.3^[a, c]^	2.5^[a, b]^
Lower secondary level completed	30.9^[b, c]^	24.0^[a, c]^	8.4^[a, b]^
Upper secondary level completed	16.0^[b]^	28.2^[a]^	22.2
Vocational level completed	4.5^[b]^	10.1^[a]^	14.2
College or higher education completed	10.1^[c]^	17.9^[c]^	52.7^[a, b]^
*Household assets, %*			
Digital TV	72.5^[b, c]^	92.7^[a]^	93.8^[a]^
Computer/laptop	13.5^[b, c]^	43.3^[a]^	40.1^[a]^
Freezer	11.5	13.0	13.6
Water heater	68.7^[b, c]^	95.7^[a, c]^	96.8^[a]^
Microwave oven	22.9^[b, c]^	62.4^[a, c]^	86.4^[a, b]^
*Household dwelling, %*			
Own a house	93.7^[c]^	93.5^[c]^	85.5^[a, b]^
Uses improved drinking water source	84.2^[b, c]^	99.8^[a]^	99.8^[a]^
Uses improved cooking fuel	63.2^[b, c]^	99.9^[a]^	100.0^[a]^
Flush and water seal toilet	88.0^[b, c]^	99.4^[a, c]^	99.7^[a, b]^
*Household food insecurity, %*			
HFIAS: food insecure	45.3^[b, c]^	10.6^[a, c]^	7.5^[a, b]^

Note: Improved drinking water source includes piped water, bottled water, and protected well/spring. Improved cooking fuel includes electricity and gas/biogas. Differences between study areas were analyzed using multilevel mixed-effect linear regression models with robust SEs and adjusted for clustering at the commune level. Superscript letters in square brackets (e.g., [a]) show the column from which the value in the current column is significantly different, *P* value < 0.05. Variables in this table are presented only for descriptive characterization of adolescents and their households.

Abbreviations: HFIAS, household food insecurity access scale; TV, television.

### Food outlet characteristics

About one-third of outlets in the rural area were convenience stores. In peri-urban areas, close to two-thirds of outlets were food stalls, stands, and tabletop vendors compared with 28% to ‒29% in rural and urban areas, respectively (Table 2). At least 1 HFG was on offer in >70% of outlets across the 3 areas. Fruits were sold in a quarter of outlets in the rural area, and in ∼15% in peri-urban and urban areas. Vegetables, on the contrary, were more available across areas (41% in rural, 47% in peri-urban, and 34% in urban). UFG were widely available in outlets in the rural and the urban areas (>70%), with ∼60% of the outlets selling UPF and half selling SSBs. By contrast, in peri-urban areas, less than one-third of outlets offered UPF, 20% offered SSB, and 48% offered UFG.

**TABLE 2:** Outlet characteristics by geographical location

	Geographical location		
	Rural [a]	Peri-urban [b]	Urban [c]
	*n* = 1151	*n* = 1213	*n* = 3478
**Outlet type, %**			
Supermarket	0.1	0.1	0.1
Convenience store (part of a chain)	0.3^[b, c]^	0.5^[a, c]^	1.1^[a, b]^
Convenience store (not part of a chain)	28.6^[b, c]^	6.3^[a]^	7.9^[a]^
Store primarily selling non-food items/services	4.7^[b, c]^	1.6^[a]^	2.0^[a]^
Food stall/stand/tabletop inside toad market	5.1^[c]^	7.9	17.0^[a]^
Food and beverage stall/stand/tabletop	28.2^[b]^	63.3^[a, c]^	28.5^[b]^
Mobile vendor	5.4	6.3	5.2
Bakery/pastry shop	1.0	0.7	0.9
Fast food restaurant: Western style	0.2	0.3	0.4
Non–fast-food restaurant	12.8^[b, c]^	3.6^[a, c]^	17.5^[a, b]^
Coffee/fresh juice shop	1.9^[b, c]^	3.1^[a, c]^	10.9^[a, b]^
Dairy shop	1.7^[b, c]^	0.2^[a]^	0.1^[a]^
Bar, pub	0.2^[b, c]^	1.7^[a, c]^	0.6^[a, b]^
Bubble tea store	0.7	0.5^[c]^	1.2^[b]^
Nonconvenience food store	9.0	3.5^[c]^	6.4^[b]^
Other	0.0^[c]^	0.1	0.1^[a]^
**Proportion of outlets selling specific food groups, %**			
HFG	77.0^[c]^	77.2^[c]^	70.5^[a, b]^
Fruits	26.1^[b, c]^	15.3^[a]^	14.5^[a]^
Vegetables	40.8^[c]^	47.1^[c]^	34.0^[a, b]^
UFG	70.5^[b]^	47.8^[a, c]^	78.4^[b]^
SSB	52.1^[b]^	19.4^[a, c]^	47.0^[b]^
UPF	58.9^[b]^	29.8^[a, c]^	60.4^[b]^
UXFG	57.3^[b, c]^	26.9^[a, c]^	46.5^[a, b]^

Note: A toad market in the Vietnamese context refers to an open space that is not officially recognized as a market. They could be located just outside a (wet) market or not. SSB is defined using the definition by the WHO. Differences were analyzed using mixed-effects linear regression models with robust estimation of SEs to adjust for clustering at the commune level. Superscript letters in square brackets (e.g., [a]) show the column from which the value in the current column is significantly different, *P* value < 0.05.

Abbreviations: HFG, healthy food group; SSB, sugar-sweetened beverage; UFG, unhealthy food group; UPF, ultraprocessed food; UXFG, unhealthy in excessive amounts food group.

### Food environment assessment

#### Physical distance to food outlets and food groups sold around adolescents’ homes and schools by urbanicity

**FIGURE 1:**
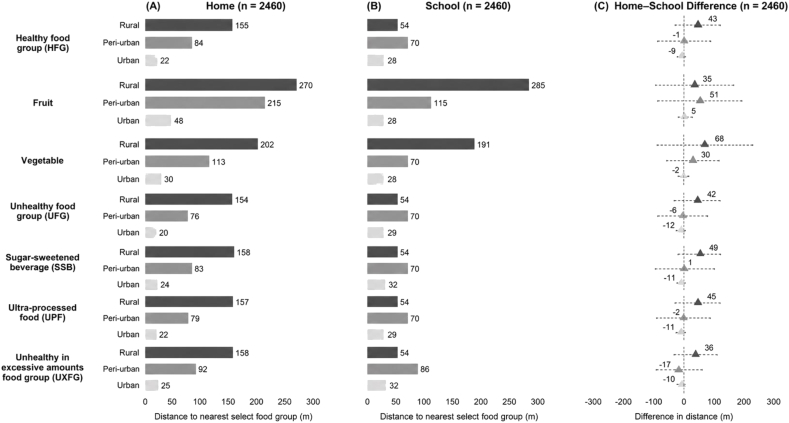
Median distance to the nearest select food group around adolescents’ homes and schools by geographical location. Note: SSB is defined using the definition by the WHO. Values are median and rounded to the nearest point.

Proximity to food outlets when at home was highest for adolescents living in urban areas, followed by those in peri-urban areas, and the rural area (Figure 1 and Supplementary Table 3). Similar patterns were observed for both healthy (HFG, fruits, and vegetables) and unhealthy (UFG, SSB, UPF, and UXFG) food groups (Figure 1). For example, the nearest SSB and UPF were only ∼25 m away from home for urban adolescents, compared with 150 m in the rural area. Most of the UPFs were SSB, explaining the similar results for these 2 food categories (Figure 1). Fruits were ∼25 m further away from home (as compared with SSB and UPF) in urban areas; and in the peri-urban and rural settings, the distance was ≥100 m longer. Urban adolescents also had significantly closer access to HFG, UFG, and UXFG compared with their peers living in peri-urban and rural areas. Compared with other food groups, fruits and vegetables were the furthest from home in all 3 areas. The pattern was different for the other healthy foods included in the HFG, which tended to be within similar distances as UFG.

A different pattern was observed for proximity to food when at school (Supplementary Table 3). For urban adolescents, all types of foods were <∼50 m away. HFG, UFG, SSB, and UPF were also found very close to schools in the rural area (<54 m) but were slightly further away in the peri-urban area (∼70 m). Fruits were ∼200 m further away from school (as compared with SSB and UPF) in the rural area; in the peri-urban area, the distance was ≥40 m longer, whereas in the urban area it was only ∼10 m. When in school, adolescents in the rural and the urban settings had to walk a shorter distance than those in the peri-urban area to access HFG and UFG.

#### Density of food outlets and specific food groups around adolescents’ homes and schools by urbanicity

**FIGURE 2:**
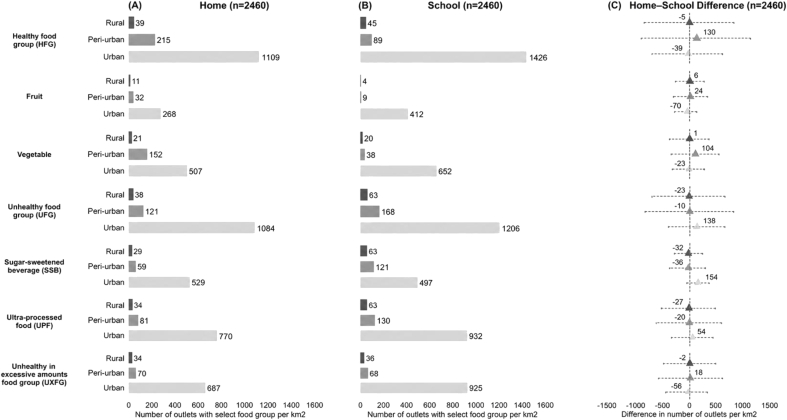
Mean number of outlets per square kilometer with select food groups within a 100-m radius of adolescents’ homes and schools by geographical location. Note: SSB is defined using the definition by the WHO. Values are mean and rounded to the nearest point. The number of outlets within the 100-m radius can be calculated as follows. The surface area within the 100-m radius equals (0.1 km)^2^ x π or 0.031416 km^2^. A density of 1109 outlets/km^2^ equals a total of 1109 outlets/km^2^ × 0.031416 km^2^ or ∼35 outlets within the 100-m buffer.

The density of outlets selling food in the urban area was higher than in the peri-urban and rural areas, respectively (by ∼5 and ∼30 times in home and school environments) (Supplementary Table 4). Availability of outlets selling HFG as well as those selling UFG or UXFG was also significantly higher for urban adolescents compared with their peers living in peri-urban and rural areas (Figure 2). The relative availability of specific food groups varied across study sites and between home and school environments, with no consistent pattern emerging (Supplementary Table 4).

#### Diversity of food group offerings around adolescents’ homes and schools by urbanicity

**FIGURE 3:**
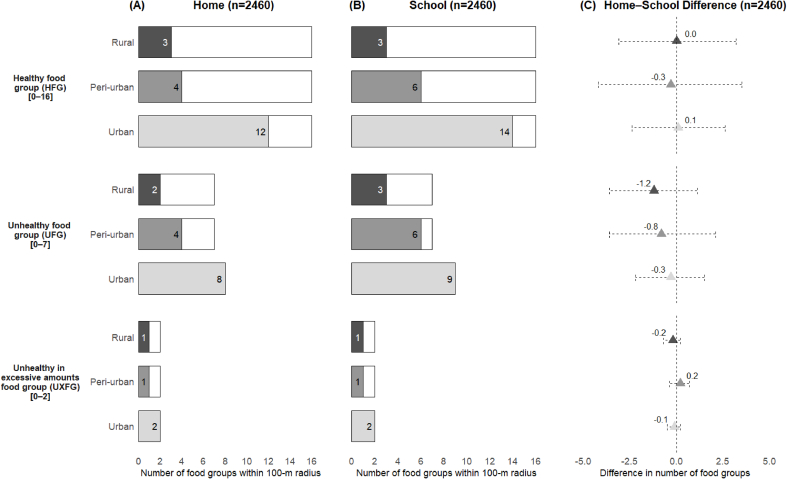
Food group diversity within a 100-m radius of adolescent home/school by environment and geographical location. Note: Food group diversity measured as unique food group count. Values are mean and rounded to the nearest point. The outline indicates the potential range of food groups: HFG is 0‒16; UFG is 0‒7, and UXFG is 0‒2. HFG, healthy food group; UFG, unhealthy food group; UXFG, unhealthy in excessive amounts food group.

Adolescents in the urban area had access to (almost) all HFG (∼12/16 groups), UFG (∼6/7), and UXFG (2/2) within 100 m of their home and school, as compared with peri-urban and rural adolescents, who had much less diversity (∼3‒6/16 HFG; ∼3/7 UFG; 1/2 UXFG) (Figure 3 and Supplementary Table 5).

#### Differences between adolescents’ home and school food environments

We did not find evidence that school food environments were unhealthier than home food environments within each study site. The proximity, density, and diversity measures used were the same for the home and school food environments in all study sites (FIGURE 1, FIGURE 2, FIGURE 3 and Supplementary Tables 6‒8). Although the point estimates suggested noticeable differences between home and school food environments (particularly in rural areas for several food groups including HFG, UFG, SSB, UPF, and UXFG), these estimates were not significantly different from 0, indicating no clear evidence of systematic differences.

#### Sensitivity analyses

Sensitivity analyses using KDE confirmed the robustness of our findings, with results consistently showing a higher concentration of food outlets within a 100-m radius from adolescents’ homes and schools in urban areas compared with peri-urban and rural areas (Supplementary Table 9). The highest KDE was found for outlets selling HFG in the urban area, with the lowest KDE for outlets selling fruits in the rural area. KDE for HFG, UFG, and UXFG were higher in the urban area as compared with peri-urban and rural areas.

We did not observe any difference in KDE between adolescents’ home and school environments (Supplementary Table 10).

The overall conclusions remained unchanged using different radii (200 m and 400 m), with urban households and schools having greater availability of all types of foods (Supplementary Tables 11 and 12). Similarly, results were robust after excluding from analyses households or schools located <100 m from the commune boundary (results not shown).

## Discussion

Our study provides a comprehensive assessment of adolescents’ home and school food environments across the rural-urban continuum in Northern Vietnam.

Our first objective was to assess the healthiness of the food environment across the 3 study sites, which differed by urbanicity. We compared distance, density, and diversity of healthy and unhealthy foods around home and school, by area of residence. When at home, adolescents living in urban households had greater access to both healthy and unhealthy food options compared with those living in the rural or the peri-urban area. Fruit and vegetables were generally the furthest and least available food groups, but differences in distance between food group categories were small (mostly <100 m) and unlikely to affect accessibility. We also found that foods from the HFG, including whole grains, eggs, fish, low-fat dairy, and legumes, in addition to fruits and vegetables, were available within similar distances as foods from the UFG from adolescents’ homes. In school environments, both healthy and unhealthy foods were available within very short walking distances across study sites, although fruit and vegetables tended to be slightly further away. A clear rural-urban gradient was observed in density measures both around home and school, with markedly higher densities of all food groups in urban compared with the peri-urban and rural areas. Similarly, another study conducted in Vietnam found urban outlet densities to be 3 to 4 times higher than those from peri-urban and rural areas [19]. This finding reflects underlying differences in population density across these contexts.

Our second goal was to assess whether school food environments were unhealthier than home food environments. Contrary to widespread concern that school food environments expose adolescents to particularly unhealthy foods [15,16,34], our analyses show no evidence that school food environments were less healthy than those surrounding the home. Within each setting, adolescents’ exposure to foods of varying healthfulness was broadly similar. It is worth noting that our conclusion is not that school environments were healthy in our study settings but rather that they were not different from the home food environments. The distance point estimates in the rural setting were ≥3 times higher for the home as compared with the school environment. This large difference reflects the location of schools near the main road and in town centers surrounded by food outlets, whereas adolescents’ homes were distributed both centrally and more peripherally. In urban and peri-urban areas, the home-to-outlet and school-to-outlet distance point estimates were similar given that adolescents’ homes and schools are embedded within the same commercial landscapes, characterized by widespread availability of both healthy and unhealthy foods.

A key question is whether the urban-rural differences in food environment translate into differences in adolescent diets. Our assessment of the healthiness of adolescent diets in the same study sample found no meaningful differences in diet healthiness by urbanicity [20]. The absence of a clear association between food environment characteristics and diets suggests that, within the range of food environment variations observed, differential exposure may not be a dominant driver of what adolescents eat. It is important to note that in our sample, only 25% of the adolescents’ energy intake came from foods prepared outside the home [35]. It is therefore possible that the influence of food environments on adolescent consumption patterns would be more pronounced in contexts where adolescents receive more pocket money and have more opportunities to purchase foods outside the home.

This finding should not be interpreted as evidence that food environments are irrelevant. Rather, it points to a context in which all 3 settings provide ample access to both healthy and unhealthy food options. In these contexts, whether outlets are located 25 m or 250 m away may have limited practical significance for consumption. Adolescents appear willing and able to access these foods, either near home or near school (G. Fretes, P.H. Nguyen, S. Gune, K. Maasen, E.F. Talsma, M.T. Truong, et al., unpublished results, 2025). However, preferences, affordability, social norms, marketing, and actual interaction with the food environment may play a more prominent role in shaping diets than spatial variation in outlet location. Among the same study sample, we found that more direct engagement with the food environment (i.e., defined as consuming foods prepared outside the home) was associated with a lower healthiness of adolescent diets. This pattern was consistent across the rural, peri-urban and urban settings, suggesting that even when healthier options are available in the food environment, adolescents disproportionately choose unhealthier foods (G. Fretes, P.H. Nguyen, S. Gune, K. Maasen, E.F. Talsma, M.T. Truong, et al., unpublished results, 2025). A systematic review of the literature suggests that greater accessibility to and availability of foods in the food environment in LMICs was associated with diets [7], although the evidence on specific dietary outcomes remained inconsistent. The review also reported that in some studies, greater availability and access to healthy foods like fruits and vegetables were found to be associated with intake. These findings do not establish that higher availability and access increase demand, as higher demand for these foods may also shape the distribution of outlets supplying them. The magnitude and mechanisms of these relationships remain poorly understood and warrant additional research in other LMIC countries and contexts.

Could our findings be due to methodological limitations? Even though we collected data on every outlet within the study areas, homes or schools near the boundary had access to outlets not captured in our survey. We normalized the density calculations to prevent underestimation due solely to differences in the observable area size. This does not rule out, however, potential measurement errors due to households or schools being located near the boundary for which outlets outside the area were not surveyed. We analyzed sensitivity analyses excluding households and schools located within 100 m of the commune boundary, and results were robust, with only small changes in magnitude and no changes in the significance that could affect our conclusions (results not shown). To assess the robustness of our findings to the use of a sharp 100-m radius buffer, we conducted sensitivity analyses using alternative radii and KDE, which integrates outlet proximity and concentration without imposing hard buffer boundaries. Results were generally consistent across these alternative specifications, supporting the robustness of our conclusions.

A major strength of our paper is the comprehensive assessment of the home and school food environments, which allowed direct comparisons. Previous studies that emphasized schools as critical sites of unhealthy food exposure [[15], [16], [17],^34^] have typically lacked comparisons with home environments. Exposure to foods (healthy or unhealthy) accumulates across home, school, and between them, and research that fails to account for this complexity risks overestimating the influence of any single environment.

### Policy implications

In our study, healthy and unhealthy foods were similarly accessible around both home and school, indicating that adolescents encounter similar food environments across their main activity spaces. Most adolescents lived close to school and typically walked or used a bicycle or scooter to commute, giving them easy access to food outside the immediate school setting. This suggests that policies focused solely on improving the school food environment may have limited impact on the overall healthiness of adolescent diets in this context. Moreover, the relatively small differences we observed in distance and density measures across rural, peri-urban, and urban areas, combined with the absence of differences in adolescent diets across the same settings, suggest that policies that restrict (rather than eliminate) availability and access are unlikely to yield meaningful dietary improvements in Northern Vietnam. Once unhealthy foods are available in the food environment, even relatively limited access may be sufficient to prompt consumption.

Given that both healthy and unhealthy foods are available everywhere in the Vietnamese environment studied, policies should prioritize making healthy foods the easier and most appealing choice. Improving the healthiness of adolescent diets will require a combination of interventions, including the introduction of easy-to-understand front-of-package labeling, taxation of unhealthy foods and beverages, communication campaigns directed to adolescents, and restrictions on the marketing of unhealthy foods across all media platforms, including digital media. Additional strategies such as point-of-purchase nudges to enhance the visibility of healthy foods and the implementation of school meal standards will also be needed. Experiences from other LMICs and regions, including several countries in Latin America, could provide valuable insights to guide the development of context-specific and effective interventions in Vietnam.

## Author contributions

The authors’ responsibilities were as follows – GF, IDB, MTR, JLL: designed the research; GF, NTZ, PHN, ALP, KM, EFT, MTT, TTTT, IDB, MTR, JLL: conducted the research and interpreted the data; NTZ: analyzed and interpreted the data; GF: wrote the paper; GF, NTZ, PHN, ALP, KM, EFT, MTT, TTTT, IDB, MTR, JLL: provided the critical revision of the manuscript for important intellectual content; and all authors: read and approved the final manuscript.

## Data availability

Data described in the manuscript, code book, and analytic code will be made available upon request.

## Declaration of AI and AI-assisted Technologies in the Writing Process

The authors declare that no generative AI or AI-assisted technologies were used in the writing of this manuscript.

## Funding

This study was part of the Consultative Group on International Agricultural Research (CGIAR)-funded Sustainable Healthy Diets through Food Systems Transformation research initiative, which aims to ensure sustainable healthy diets for all by stimulating the demand for sustainable healthy diets and the supply of sustainable nutritious foods, while also improving livelihoods, gender equity, and social inclusiveness in all sectors of food systems. The funders had no role in study design, data collection and analysis, data interpretation, decision to publish, or preparation of the manuscript.

## Conflict of interest

The authors report no conflicts of interest.
