# Supplementary material for: Food Is Always Close By: An Assessment of the Healthiness of the Adolescent Home and School Food Environments in Northern Vietnam
Source: Curr Dev Nutr. 2026 Jun 17;10(7):109402. doi: 10.1016/j.cdnut.2026.109402 (PMC13381996; doi:10.1016/j.cdnut.2026.109402)
Supplement: Multimedia component 1 [file mmc1.docx]

**Supplementary Table 1. Food groups based on the Global Dietary Quality Score (GDQS) classification**

|  | **GDQS food group** |
| --- | --- |
| Healthy food groups (HFG) | Citrus fruit |
|  | Deep orange fruits |
|  | Other fruits |
|  | Dark green leafy vegetables |
|  | Cruciferous vegetables |
|  | Deep orange vegetables |
|  | Other vegetables |
|  | Legumes |
|  | Deep orange tubers |
|  | Nuts and seeds |
|  | Whole grains |
|  | Liquid oils |
|  | Fish and shellfish |
|  | Poultry and game meat |
|  | Low fat dairy |
|  | Eggs |
| Unhealthy food groups (UFG) | Processed meat |
|  | Refined grains and baked goods |
|  | Sweets and ice cream |
|  | Sugar-sweetened beverages |
|  | Juice |
|  | White roots and tubers |
|  | Purchased deep fried foods |
| Unhealthy in excessive amounts food group (UXFG) | High fat dairy  Red meat |

Reference: Adapted from Bromage S, Batis C, Bhupathiraju SN, Fawzi WW, Fung TT, Li Y, Deitchler M, Angulo E, Birk N, Castellanos-Gutiérrez A, et al. Development and Validation of a Novel Food-Based Global Diet Quality Score (GDQS). J Nutr. 2021;151:75S-92S.

**Supplementary Table 2. Outlet typology definitions**

| **Type of outlet** | **Definition** |
| --- | --- |
| Supermarket | A shop that sells a multitude of products (food as well as nonfood) in a closed-off environment where customers can freely walk around and check and choose the products that they want to purchase using baskets or trolleys. e.g., Winmart, Unik Mart, etc. |
| Convenience store (part of a chain) | This category is limited to those shops that are part of a chain. E.g., Winmart +, Circle K, etc. |
| Convenience store (not part of a chain) | A store that could sell other items, but the primary focus is on selling food in a closed-off environment (the size of the store does not matter) |
| Store primarily selling non-food-items/services | This category includes stores that do not sell food as part of their main business but as a complement. e.g., agriculture stores, supply stores, travel agencies, etc. |
| Food stall/stand/tabletop inside toad market | Includes vendors that assemble in an open space that is not officially recognized as a market. They could be located just outside a (wet) market or not |
| Food stall/stand/tabletop | Outlets that do not have four permanent walls but typically do not move during the day |
| Mobile vendor | Vendors who sell food out of a moveable vehicle (truck, cart, trailer, motorcycle, bicycle) or from a basket, box, etc. they carry |
| Bakery/pastry shop | A place that primarily sells bread, cakes, and pastries. |
| Fast food restaurant | Western style. e.g., McDonald’s, KFC, Lotteria, Pizza Company |
| Non-fast food restaurant |  |
| Coffee/fresh juice shop | A shop that primarily sells coffee, tea, and sweet snacks/desserts |
| Dairy shop | A shop that primarily sells ice cream, frozen yogurt, and (sweetened) dairy beverages |
| Bar/pub | A shop that primarily sells (alcoholic) beverages |
| Bubble tea store | A shop that primarily sells bubble tea and tea drinks |
| Non-convenience food store | Specialized food stores. E.g., fruit store, butcher |
| Other | Only if cannot be otherwise listed |

**Supplementary Table 3. Proximity to nearest food outlet and select food groups around adolescents’ home and school by geographical location**

|  | | **Home food environment** | | | **School food environment** | | | | |
| --- | --- | --- | --- | --- | --- | --- | --- | --- | --- |
|  | **Rural**  **[a]** | | **Peri-urban**  **[b]** | **Urban**  **[c]** | **Rural** | | **Peri-urban** | | **Urban** |
|  | **Median**  **(IQR)** | | **Median**  **(IQR)** | **Median**  **(IQR)** | **Median**  **(IQR)** | | **Median**  **(IQR)** | | **Median**  **(IQR)** |
|  | n = 1,067 | | n = 933 | n = 663 | n = 5 | n = 3 | | n = 4 | |
| **Distance to nearest**  **food outlet, m** | 145.1 (210.3) ^[b, c]^ | | 69.5 (82.2) ^[a, c]^ | 19.2 (25.6) ^[a, b]^ | 54.1 (162.6) | 70.2 (153.2) | | 38.1 (36.1) | |
| **Distance to nearest**  **food group, m** |  | |  |  |  |  | |  | |
| HFG | 154.6 (211.1) ^[b, c]^ | | 82.3 (95.5) ^[a, c]^ | 22.4 (27.5) ^[a, b]^ | 54.1 (162.6) | 70.2 (171.7) | | 38.1 (36.1) | |
| Fruits | 270.0 (496.8) ^[c]^ | | 216.3 (236.0) ^[c]^ | 47.7 (59.6) ^[a, b]^ | 285.3 (77.7) | 114.7 (248.7) | | 51.3 (122.3) | |
| Vegetables | 202.3 (335.4) ^[b, c]^ | | 113.2 (122.0) ^[a, c]^ | 30.1 (34.4) ^[a, b]^ | 191.1 (118.0) | 70.2 (171.7) | | 38.1 (41.7) | |
| UFG | 154.3 (214.9) ^[b, c]^ | | 75.7 (84.7) ^[a, c]^ | 19.9 (26.3) ^[a, b]^ | 54.1 (162.6) | 70.2 (164.4) | | 38.8 (35.4) | |
| SSB | 157.6 (223.4) ^[b, c]^ | | 82.0 (94.0) ^[a, c]^ | 24.0 (28.2) ^[a, b]^ | 54.1 (162.6) | 70.2 (171.7) | | 40.0 (32.9) | |
| UPF | 156.7 (220.2) ^[b, c]^ | | 78.4 (85.7) ^[a, c]^ | 21.7 (27.5) ^[a, b]^ | 54.1 (162.6) | 70.2 (164.4) | | 38.8 (35.4) | |
| UXFG | 158.0 (217.0) ^[b, c]^ | | 90.3 (97.8) ^[a, c]^ | 25.1 (27.8) ^[a, b]^ | 90.4 (154.3) | 85.7 (128.6) | | 40.0 (34.3) | |

Note: HFG: healthy food group. UFG: unhealthy food group. UXFG: unhealthy in excessive amounts food group. SSB: sugar-sweetened beverages using the definition by the World Health Organization (WHO). UPF: ultra-processed foods. Values are winsorized at 95^th^ percentile and presented as median (IQR), where IQR is the interquartile range calculated as the difference between the 75th and 25th percentiles. Differences between study areas were analyzed using mixed-effects quantile regression models with robust estimation of standard errors to adjust for clustering at the commune level. Superscript letters in square brackets (e.g., [a]) show the column from which the value in the current column is significantly different, *P value* < 0.05. Given the small sample size, no statistical testing of differences in the school food environment by study site was conducted.

**Supplementary Table 4. Density of food outlets with select food groups within 100-meter radius of adolescents’ home and school by geographical location**

|  | **Home food environment (100-m radius)** | | | **School food environment (100-m radius)** | | |
| --- | --- | --- | --- | --- | --- | --- |
|  | **Rural**  **[a]** | **Peri-urban**  **[b]** | **Urban**  **[c]** | **Rural** | **Peri-urban** | **Urban** |
|  | **Mean** ± **SD** | **Mean** ± **SD** | **Mean** ± **SD** | **Mean** ± **SD** | **Mean** ± **SD** | **Mean** ± **SD** |
|  | n = 1,067 | n = 933 | n = 663 | n= 5 | n= 3 | n= 4 |
| **Absolute density** |  |  |  |  |  |  |
| Number of outlets per km^2^ | 49.0 ± 134.1 ^[b, c]^ | 262.6 ± 816.8 ^[a, c]^ | 1553.1 ± 1298.7 ^[a, b]^ | 57.3 ± 65.3 | 180.5 ± 175.4 | 1687.8 ± 1730.0 |
| **Number of outlets with**  **food group per km^2^*** |  |  |  |  |  |  |
| HFG | 38.7 ± 103.3 ^[b, c]^ | 206.1 ± 685.5 ^[a, c]^ | 1109.4 ± 1001.4 ^[a, b]^ | 44.6 ± 53.3 | 95.5 ± 95.5 | 1313.6 ± 1333.8 |
| Fruits | 10.5 ± 35.8 ^[b, c]^ | 32.0 ± 121.9 ^[a, c]^ | 268.3 ± 339.2 ^[a, b]^ | 6.4 ± 14.2 | 10.6 ± 18.4 | 374.2 ± 396.4 |
| Vegetables | 20.9 ± 55.3 ^[b, c]^ | 137.9 ± 573.3 ^[a, c]^ | 506.5 ± 433.1 ^[a, b]^ | 19.1 ± 28.5 | 42.5 ± 48.6 | 613.0 ± 562.1 |
| UFG | 37.9 ± 92.6 ^[b, c]^ | 126.3 ± 255.3 ^[a, c]^ | 1084.4 ± 674.6 ^[a, b]^ | 51.0 ± 57.9 | 180.5 ± 175.4 | 1130.5 ± 1087.6 |
| SSB | 29.2 ± 61.1 ^[b, c]^ | 59.7 ± 86.9 ^[a, c]^ | 528.7 ± 299.2 ^[a, b]^ | 51.0 ± 57.9 | 127.4 ± 114.8 | 485.6 ± 383.4 |
| UPF | 33.8 ± 81.1 ^[b, c]^ | 84.7 ± 139.8 ^[a, c]^ | 769.7 ± 445.2 ^[a, b]^ | 51.0 ± 57.9 | 138.0 ± 128.7 | 875.8 ± 792.3 |
| UXFG | 33.6 ± 80.7 ^[b, c]^ | 74.8 ± 147.4 ^[a, c]^ | 686.7 ± 490.2 ^[a, b]^ | 31.8 ± 31.8 | 74.3 ± 80.1 | 859.8 ± 828.4 |

Note: HFG: healthy food group. UFG: unhealthy food group. UXFG: unhealthy in excessive amounts food group. SSB: sugar-sweetened beverages using the definition by the World Health Organization (WHO). UPF: ultra-processed foods. Values are mean (SD). Differences in densities between study areas were analyzed using mixed effects Poisson regression models with robust estimation of standard errors to adjust for clustering at the commune level and are expressed as relative risks (RR). Superscript letters in square brackets (e.g., [a]) show the column from which the value in the current column is significantly different, *P value* < 0.05. Given the small sample size, no statistical testing of differences in the school food environment by study site was conducted.

**Supplementary Table 5. Food group diversity around adolescents’ home and school by geographical location**

|  | **Home food environment**  **(100-meter radius)** | | | **School food environment**  **(100-meter radius)** | | |
| --- | --- | --- | --- | --- | --- | --- |
|  | **Rural**  **[a]** | **Peri-urban**  **[b]** | **Urban**  **[c]** | **Rural** | **Peri-urban** | **Urban** |
|  | **Mean** ± **SD** | **Mean** ± **SD** | **Mean** ± **SD** | **Mean** ± **SD** | **Mean** ± **SD** | **Mean** ± **SD** |
|  | n = 1,067 | n = 933 | n = 663 | n = 5 | n = 3 | n = 4 |
| **Food group diversity*** |  |  |  |  |  |  |
| HFG [potential range 0 - 16] | 2.5 ± 4.0 ^[b, c]^ | 4.2 ± 4.8 ^[a, c]^ | 12.4 ± 3.8 ^[a, b]^ | 2.6 ± 3.8 | 6.3 ± 7.1 | 12.5 ± 3.7 |
| UFG [0 - 7] | 1.5 ± 2.1 ^[b, c]^ | 2.6 ± 2.4 ^[a, c]^ | 6.2 ± 1.6 ^[a, b]^ | 2.4 ± 2.2 | 4.7 ± 4.0 | 6.5 ± 0.6 |
| UXFG [0 - 2] | 0.5 ± 0.8 ^[b, c]^ | 0.9 ± 0.9 ^[a, c]^ | 1.9 ± 0.4 ^[a, b]^ | 0.8 ± 0.8 | 1.0 ± 1.0 | 2.0 ± 0.0 |

Note: *Unique food group count. HFG: healthy food group. UFG: unhealthy food group. UXFG: unhealthy in excessive amounts food group. Potential range of HFG is 0-16; UFG is 0-7, and UXFG is 0-2. Values are mean (SD). Differences were analyzed using mixed-effects linear regression models with robust estimation of standard errors to adjust for clustering at the commune level. Superscript letters in square brackets (e.g., [a]) show the column from which the value in the current column is significantly different, *P value* < 0.05. Given the small sample size, no statistical testing of differences in the school food environment by study site was conducted.

**Supplementary Table 6. Differences in proximity to nearest food outlet and select food groups between adolescents’ home and school food environments**

|  | **Rural** | | | **Peri-urban** | | | **Urban** | | |
| --- | --- | --- | --- | --- | --- | --- | --- | --- | --- |
|  | **Home**  **[a]** | **School**  **[b]** | **Δ between home-school** | **Home**  **[a]** | **School**  **[b]** | **Δ between home-school** | **Home**  **[a]** | **School**  **[b]** | **Δ between home-school** |
|  | **Median (IQR)** | **Median (IQR)** | **Δ**  **(95% CI)** | **Median (IQR)** | **Median (IQR)** | **Δ**  **(95% CI)** | **Median (IQR)** | **Median (IQR)** | **Δ**  **(95% CI)** |
|  | n = 1,067 | n = 1,067 |  | n = 730 | n = 730 |  | n = 663 | n = 663 |  |
| **Distance to nearest food outlet, m** | 145.1 (210.3) | 54.1 (165.5) | 35.9 | 70.2 (85.3) | 70.2 (153.2) | -8.1 | 19.2 (25.6) | 27.9 (25.2) | -12.1 |
|  |  |  | (-41.7, 113.4) |  |  | (-78.9, 62.8) |  |  | (-27.6, 3.5) |
| **Distance to nearest food group, m** |  |  |  |  |  |  |  |  |  |
| HFG | 154.6 (211.1) | 54.1 (165.5) | 42.7 | 83.7 (98.8) | 70.2 (171.7) | -0.9 | 22.2 (27.5) | 27.9 (25.2) | -9.0 |
|  |  |  | (-30.2, 115.6) |  |  | (-89.4, 87.6) |  |  | (-23.0, 5.1) |
| Fruits | 270.0 (496.8) | 285.3 (77.7) | 34.9 | 215. (255.0) | 114.7 (248.7) | 50.8 | 47.7 (59.6) | 27.9 (46.9) | 5.4 |
|  |  |  | (-94.4, 164.2) |  |  | (-89.0, 190.5) |  |  | (-15.6, 26.4) |
| Vegetables | 202.3 (335.4) | 191.1 (154.1) | 67.9 | 113.0 (125.3) | 70.2 (171.7) | 29.9 | 30.1 (34.4) | 27.9 (36.3) | -1.8 |
|  |  |  | (-89.5, 225.3) |  |  | (-55.8, 115.5) |  |  | (-20.0, 16.4) |
| UFG | 154.3 (214.9) | 54.1 (165.5) | 42.5 | 76.3 (87.2) | 70.2 (164.4) | -5.8 | 19.9 (26.3) | 29.3 (23.8) | -12.4 |
|  |  |  | (-33.9, 118.9) |  |  | (-88.0, 76.4) |  |  | (-27.2, 2.5) |
| SSB | 157.6 (223.4) | 54.1 (165.5) | 48.8 | 83.3 (101.2) | 70.2 (171.7) | 0.9 | 24.0 (28.2) | 31.6 (21.4) | -11.0 |
|  |  |  | (-20.5, 118.0) |  |  | (-97.5, 99.3) |  |  | (-25.8, 3.9) |
| UPF | 156.7 (220.2) | 54.1 (165.5) | 44.6 | 79.4 (92.7) | 70.2 (164.4) | -1.6 | 21.7 (27.5) | 29.3 (23.8) | -11.3 |
|  |  |  | (-30.6, 119.8) |  |  | (-92.4, 89.1) |  |  | (-26.7, 4.1) |
| UXFG | 158.0 (217.0) | 54.1 (154.3) | 35.9 | 92.3 (102.3) | 85.7 (128.6) | -17.1 | 25.1 (27.8) | 31.6 (21.4) | -9.6 |
|  |  |  | (-34.2, 106.0) |  |  | (-92.7, 58.5) |  |  | (-20.5, 1.4) |

Note: HFG: healthy food group. UFG: unhealthy food group. UXFG: unhealthy in excessive amounts food group. SSB: sugar-sweetened beverages using the definition by the World Health Organization (WHO). UPF: ultra-processed foods. Values are winsorized at 95^th^ percentile and presented as median (IQR), where IQR is the interquartile range calculated as the difference between the 75th and 25th percentiles. Differences between environments were analyzed using multilevel mixed-effect quantile regression models with robust estimation of standard errors to adjust for clustering at the commune level.

**Supplementary Table 7. Differences in density of food outlets with select food groups within 100-m radius between adolescents’ home and school food environments**

|  | **Rural** | | | **Peri-urban** | | | **Urban** | | |
| --- | --- | --- | --- | --- | --- | --- | --- | --- | --- |
|  | **Home**  **(100-m radius)**  **[a]** | **School**  **(100-m radius)**  **[b]** | **Δ between home-school** | **Home**  **(100-m radius)**  **[a]** | **School**  **(100-m radius)**  **[b]** | **Δ between home-school** | **Home**  **(100-m radius)**  **[a]** | **School**  **(100-m radius)**  **[b]** | **Δ between home-school** |
|  | **Mean** ± **SD** | **Mean** ± **SD** | **Δ**  **(95% CI)** | **Mean** ± **SD** | **Mean** ± **SD** | **Δ**  **(95% CI)** | **Mean** ± **SD** | **Mean** ± **SD** | **Δ**  **(95% CI)** |
|  | n = 1,067 | n = 1,067 |  | n = 730 | n = 730 |  | n = 663 | n = 663 |  |
| **Absolute density** |  |  |  |  |  |  |  |  |  |
| Number of outlets per km^2^ | 49.0 ± 134.1 | 67.7 ± 60.1 | -16.2 | 273.2 ± 885.3 | 168.3 ± 139.2 | 123.0 | 1553.1 ± 1298.7 | 1817.8 ± 1477.0 | 74.2 |
|  |  |  | (-1089.9, 1057.5) |  |  | (-1192.1, 1438.1) |  |  | (-763.7, 912.2) |
| **Number of outlets with food group per km^2^** |  |  |  |  |  |  |  |  |  |
| HFG | 38.7 ± 103.3 | 45.0 ± 42.7 | -5.4 | 215.0 ± 743.9 | 88.6 ± 75.5 | 129.8 | 1109.4 ± 1001.5 | 1425.7 ± 1134.8 | -39.0 |
|  |  |  | (-838.9, 828.1) |  |  | (-891.1, 1150.7) |  |  | (-689.6, 611.7) |
| Fruits | 10.5 ± 35.8 | 4.4 ± 11.0 | 6.0 | 32.2 ± 127.9 | 8.9 ± 14.3 | 24.3 | 268.3 ± 339.2 | 412.2 ± 331.1 | -69.6 |
|  |  |  | (-249.4, 261.5) |  |  | (-288.5, 337.2) |  |  | (-268.8, 129.7) |
| Vegetables | 20.9 ± 55.3 | 20.2 ± 22.7 | 1.2 | 151.7 ± 636.4 | 38.4 ± 38.0 | 103.5 | 506.5 ± 433.1 | 652.3 ± 477.3 | -22.8 |
|  |  |  | (-362.0, 364.5) |  |  | (-341.4, 548.5) |  |  | (-308.3, 262.7) |
| UFG | 37.9 ± 92.6 | 63.3 ± 56.6 | -23.0 | 121.3 ± 252.9 | 168.3 ± 139.2 | -9.9 | 1084.4 ± 674.6 | 1205.9 ± 922.9 | 137.7 |
|  |  |  | (-702.7, 656.8) |  |  | (-842.5, 822.6) |  |  | (-391.2, 666.5) |
| SSB | 29.2 ± 61.1 | 63.3 ± 56.6 | -31.7 | 58.5 ± 87.8 | 121.1 ± 92.6 | -36.3 | 528.7 ± 299.2 | 496.5 ± 325.8 | 154.3 |
|  |  |  | (-298.2, 234.9) |  |  | (-362.8, 290.1) |  |  | (-53.2, 361.9) |
| UPF | 33.8 ± 81.1 | 63.3 ± 56.6 | -27.1 | 80.9 ± 137.8 | 130.0 ± 102.9 | -20.0 | 769.9 ± 445.2 | 931.7 ± 673.3 | 53.6 |
|  |  |  | (-533.9, 479.6) |  |  | (-640.7, 600.7) |  |  | (-340.4, 447.5) |
| UXFG | 33.6 ± 80.7 | 36.1 ± 29.2 | -1.6 | 69.7 ± 144.2 | 67.9 ± 62.8 | 18.2 | 686.7 ± 490.2 | 925.3 ± 702.9 | -55.8 |
|  |  |  | (-493.9, 490.7) |  |  | (-584.8, 621.2) |  |  | (-438.8, 327.2) |

Note: HFG: healthy food group. UFG: unhealthy food group. UXFG: unhealthy in excessive amounts food group. SSB: sugar-sweetened beverages using the definition by the World Health Organization (WHO). UPF: ultra-processed foods. Differences between environments were analyzed using multilevel mixed-effect linear regression models with robust standard errors and adjusted for clustering at the commune level.

**Supplementary Table 8. Differences in food group diversity within 100-m radius between adolescents’ home and school food environments**

|  | **Rural** | | | **Peri-urban** | | | **Urban** | | |
| --- | --- | --- | --- | --- | --- | --- | --- | --- | --- |
|  | **Home**  **(100-m radius)**  **[a]** | **School**  **(100-m radius)**  **[b]** | **Δ between home-school** | **Home**  **(100-m radius)**  **[a]** | **School**  **(100-m radius)**  **[b]** | **Δ between home-school** | **Home**  **(100-m radius)**  **[a]** | **School**  **(100-m radius)**  **[b]** | **Δ between home-school** |
|  | **Mean** ± **SD** | **Mean** ± **SD** | **Δ**  **(95% CI)** | **Mean** ± **SD** | **Mean** ± **SD** | **Δ**  **(95% CI)** | **Mean** ± **SD** | **Mean** ± **SD** | **Δ**  **(95% CI)** |
| **Food group diversity*** | n = 1,067 | n = 1,067 |  | n = 730 | n = 730 |  | n = 663 | n = 663 |  |
| HFG [potential range 0-16] | 2.5 ± 4.0 | 2.5 ± 2.9 | 0.0 | 4.3 ± 4.8 | 5.7 ± 5.5 | -0.3 | 12.4 ± 3.8 | 13.6 ± 2.4 | 0.1 |
|  |  |  | (-3.1, 3.2) |  |  | (-4.2, 3.5) |  |  | (-2.4, 2.6) |
| UFG [0-7] | 1.5 ± 2.1 | 2.6 ± 1.9 | -1.0 | 2.6 ± 2.4 | 4.5 ± 3.3 | -0.9 | 6.2 ± 1.6 | 6.5 ± 0.5 | -0.2 |
|  |  |  | (-2.9, 1.0) |  |  | (-3.3, 1.4) |  |  | (-1.7, 1.3) |
| UXFG [0-2] | 0.5 ± 0.8 | 0.8 ± 0.7 | -0.2 | 0.9 ± 0.9 | 0.9 ± 0.8 | 0.2 | 1.9 ± 0.5 | 2.0 ± 0.0 | -0.1 |
|  |  |  | (-0.7, 0.2) |  |  | (-0.4, 0.7) |  |  | (-0.5, 0.2) |

Note: *Unique food group count. HFG: healthy food group. UFG: unhealthy food group. UXFG: unhealthy in excessive amounts food group. Potential range of HFG is 0-16; UFG is 0-7 and UXFG is 0-2. Differences between environments were analyzed using multilevel mixed-effect linear regression models with robust standard errors and adjusted for clustering at the commune level. Superscript letters in square brackets (e.g., [a]) show the column from which the value in the current column is significantly different, *P value* < 0.05.

**Supplementary Table 9. Home and school food environment kernel density estimates (KDE) within 100-meter radius by geographical location**

|  | **Home food environment (100-m radius)** | | | **School food environment (100-m radius)** | | |
| --- | --- | --- | --- | --- | --- | --- |
|  | **Rural**  **[a]** | **Peri-urban**  **[b]** | **Urban**  **[c]** | **Rural** | **Peri-urban** | **Urban** |
|  | **Mean** ± **SD** | **Mean** ± **SD** | **Mean** ± **SD** | **Mean** ± **SD** | **Mean** ± **SD** | **Mean** ± **SD** |
|  | n=1,067 | n= 933 | n= 663 | n = 5 | n = 3 | n = 4 |
|  |  |  |  |  |  |  |
| KDE all outlets | 0.6 ± 2.0 ^[b, c]^ | 2.9 ± 9.1 ^[a, c]^ | 19.0 ± 20.1 ^[a, b]^ | 0.6 ± 0.7 | 1.5 ± 2.0 | 23.1 ± 25.3 |
| **KDE of outlets with food group** |  |  |  |  |  |  |
| HFG | 0.5 ± 1.6 ^[b, c]^ | 2.2 ± 7.4 ^[a, c]^ | 13.5 ± 15.0 ^[a, b]^ | 0.5 ± 0.5 | 0.9 ± 1.3 | 18.3 ± 19.9 |
| Fruits | 0.1 ± 0.6 ^[b]^ | 0.3 ± 1.5 ^[a]^ | 3.3 ± 5.0 | 0.0 ± 0.0 | 0.1 ± 0.2 | 5.1 ± 5.9 |
| Vegetables | 0.3 ± 0.8 ^[b, c]^ | 1.4 ± 5.2 ^[a, c]^ | 6.1 ± 6.4 ^[a, b]^ | 0.1 ± 0.2 | 0.5 ± 0.7 | 8.8 ± 9.0 |
| UFG | 0.5 ± 1.5 ^[b, c]^ | 1.5 ± 3.6 ^[a, c]^ | 13.2 ± 10.8 ^[a, b]^ | 0.6 ± 0.7 | 1.5 ± 2.0 | 15.1 ± 16.6 |
| SSB | 0.4 ± 1.0 | 0.7 ± 1.3 | 6.3 ± 4.9 | 0.6 ± 0.7 | 1.0 ± 1.2 | 6.4 ± 6.9 |
| UPF | 0.5 ± 1.3 ^[c]^ | 1.0 ± 2.1 ^[c]^ | 9.3 ± 7.2 ^[a, b]^ | 0.6 ± 0.7 | 1.2 ± 1.5 | 11.9 ± 12.6 |
| UXFG | 0.4 ± 1.3 ^[c]^ | 0.9 ± 2.3 | 8.5 ± 7.9 ^[a]^ | 0.3 ± 0.4 | 0.4 ± 0.6 | 11.7 ± 12.0 |

Note: HFG: healthy food group. UFG: unhealthy food group. UXFG: unhealthy in excessive amounts food group. SSB: sugar-sweetened beverages using the definition by the World Health Organization (WHO). UPF: ultra-processed foods. Values are mean (SD). Differences in KDE between study areas were analyzed using mixed effects Poisson regression models with robust estimation of standard errors to adjust for clustering at the commune level and are expressed as relative risks (RR). Superscript letters in square brackets (e.g., [a]) show the column from which the value in the current column is significantly different, *P value* < 0.05. Given the small sample size, no statistical testing of differences in the school food environment by study site was conducted.

**Supplementary Table 10. Difference in kernel density estimates (KDE) within 100-meter radius between adolescents’ home and school food environments**

|  | **Rural** | | | **Peri-urban** | | | **Urban** | | |
| --- | --- | --- | --- | --- | --- | --- | --- | --- | --- |
|  | **Home**  **(100-m radius)**  **[a]** | **School**  **(100-m radius)**  **[b]** | **Δ between home-school** | **Home**  **(100-m radius)**  **[a]** | **School**  **(100-m radius)**  **[b]** | **Δ between home-school** | **Home**  **(100-m radius)**  **[a]** | **School**  **(100-m radius)**  **[b]** | **Δ between home-school** |
|  | **Mean** ± **SD** | **Mean** ± **SD** | **Δ**  **(95% CI)** | **Mean** ± **SD** | **Mean** ± **SD** | **Δ**  **(95% CI)** | **Mean** ± **SD** | **Mean** ± **SD** | **Δ**  **(95% CI)** |
|  | n = 1,067 | n = 1,067 |  | n = 730 | n = 730 |  | n = 663 | n = 663 |  |
| **KDE all outlets** | 0.6 ± 2.0 | 0.8 ± 0.7 | -0.1 | 2.9 ± 9.6 | 1.3 ± 1.6 | 1.8 | 19.0 ± 20.1 | 23.9 ± 20.9 | -0.4 |
|  |  |  | (-15.1, 14.9) |  |  | (-16.6, 20.2) |  |  | (-12.1, 11.3) |
| **KDE of outlets with food group** |  |  |  |  |  |  |  |  |  |
| HFG | 0.5 ± 1.6 | 0.6 ± 0.5 | -0.1 | 2.3 ± 7.7 | 0.8 ± 1.1 | 1.5 | 13.5 ± 15.0 | 19.1 ± 16.4 | -1.8 |
|  |  |  | (-11.9, 11.8) |  |  | (-13.0, 16.0) |  |  | (-11.0, 7.5) |
| Fruits | 0.1 ± 0.6 | 0.0 ± 0.0 | 0.1 | 0.3 ± 1.6 | 0.1 ± 0.1 | 0.3 | 3.3 ± 5.0 | 5.4 ± 4.9 | -1.1 |
|  |  |  | (-3.5, 3.8) |  |  | (-4.2, 4.7) |  |  | (-4.0, 1.7) |
| Vegetables | 0.3 ± 0.8 | 0.2 ± 0.2 | 0.1 | 1.4 ± 5.6 | 0.5 ± 0.6 | 1.0 | 6.1 ± 6.4 | 9.1 ± 7.5 | -1.2 |
|  |  |  | (-5.5, 5.7) |  |  | (-5.9, 7.8) |  |  | (-5.6, 3.2) |
| UFG | 0.5 ± 1.5 | 0.7 ± 0.7 | -0.2 | 1.5 ± 3.5 | 1.3 ± 1.6 | 0.4 | 13.2 ± 10.8 | 15.2 ± 13.3 | 1.2 |
|  |  |  | (-9.4, 9.0) |  |  | (-10.8, 11.7) |  |  | (-6.0, 8.4) |
| SSB | 0.4 ± 1.0 | 0.7 ± 0.7 | -0.3 | 0.7 ± 1.3 | 0.9 ± 0.9 | 0.0 | 6.3 ± 4.9 | 6.0 ± 5.5 | 1.7 |
|  |  |  | (-3.9, 3.2) |  |  | (-4.3, 4.4) |  |  | (-1.0, 4.5) |
| UPF | 0.5 ± 1.3 | 0.7 ± 0.7 | -0.3 | 1.0 ± 2.0 | 1.0 ± 1.1 | 0.2 | 9.3 ± 7.2 | 11.8 ± 10.1 | 0.1 |
|  |  |  | (-7.3, 6.7) |  |  | (-8.4, 8.8) |  |  | (-5.4, 5.6) |
| UXFG | 0.4 ± 1.3 | 0.4 ± 0.4 | 0.0 | 0.9 ± 2.3 | 0.4 ± 0.5 | 0.6 | 8.5 ± 7.9 | 12.2 ± 10.0 | -1.3 |
|  |  |  | (-6.9, 7.0) |  |  | (-7.9, 9.2) |  |  | (-6.8, 4.1) |

Notes: HFG: healthy food group. UFG: unhealthy food group. UXFG: unhealthy in excessive amounts food group. SSB: sugar-sweetened beverages using the definition by the World Health Organization (WHO). UPF: ultra-processed foods. Values are mean (SD). Differences between food environments were analyzed using multilevel mixed-effect linear regression models with robust standard errors and adjusted for clustering at the commune level. Superscript letters in square brackets (e.g., [a]) show the column from which the value in the current column is significantly different, *P value* < 0.05.

**Supplementary Table 11. Density of food outlets around adolescents’ home and school by geographical location using different radii**

|  | **Within 200-meter radius** | | | **Within 400-meter radius** | | |
| --- | --- | --- | --- | --- | --- | --- |
|  | **Rural** | **Peri-urban** | **Urban** | **Rural** | **Peri-urban** | **Urban** |
|  | **Mean** ± **SD** | **Mean** ± **SD** | **Mean** ± **SD** | **Mean** ± **SD** | **Mean** ± **SD** | **Mean** ± **SD** |
|  |  |  |  |  |  |  |
| **Home food environment** | n = 1,067 | n = 933 | n = 663 | n = 1,067 | n = 933 | n = 663 |
| **Absolute density** |  |  |  |  |  |  |
| Number of outlets per km^2^ | 33.4 ± 75.0 | 228.1 ± 542.0 | 1363.01 ± 533.9 | 22.9 ± 44.8 | 192.2 ± 274.3 | 1222.1 ± 204.4 |
| **Number of outlets with food group per km^2^** |  |  |  |  |  |  |
| HFG | 26.5 ± 58.2 | 176.1 ± 439.9 | 968.94 ± 413.8 | 17.7 ± 33.7 | 147.5 ± 218.5 | 853.8 ± 141.0 |
| Fruits | 8.2 ± 22.1 | 31.3 ± 86.8 | 229.19 ± 139.1 | 5.7 ± 12.4 | 28.66 ± 43.44 | 192.6 ± 59.9 |
| Vegetables | 14.7 ± 31.5 | 112.9 ± 338.6 | 440.47 ± 190.7 | 9.6 ± 16.7 | 89.6 ± 158.1 | 398.6 ± 70.7 |
| UFG | 25.3 ± 49.9 | 110.1 ± 167.7 | 976.7 ± 320.9 | 16.8 ± 28.0 | 91.2 ± 92.2 | 904.9 ± 162.2 |
| SSB | 19.1 ± 31.3 | 48.6 ± 52.0 | 496.24 ± 182.6 | 12.5 ± 16.1 | 35.9 ± 29.0 | 496.1 ± 115.7 |
| UPF | 22.1 ± 41.3 | 72.2 ± 88.1 | 708.05 ± 239.4 | 14.5 ± 21.9 | 57.9 ± 50.5 | 674.1 ± 137.6 |
| UXFG | 21.9 ± 42.6 | 64.5 ± 93.1 | 600.74 ± 223.0 | 14.2 ± 22.3 | 53.9 ± 50.7 | 550.6 ± 113.4 |
|  |  |  |  |  |  |  |
| **School food environment** | n = 5 | n = 3 | n = 4 | n = 5 | n = 3 | n = 4 |
| **Absolute density** |  |  |  |  |  |  |
| Number of outlets per km^2^ | 38.2 ± 46.9 | 111.5 ± 69.4 | 1084.6 ± 963.2 | 29.1 ± 14.9 | 433.9 ± 321.5 | 1220.0 ± 133.5 |
| **Number of outlets with food group per km^2^** |  |  |  |  |  |  |
| HFG | 30.3 ± 39.9 | 74.3 ± 51.2 | 807.5 ± 727.1 | 22.3 ± 9.7 | 352.9 ± 271.1 | 850.3 ± 70.4 |
| Fruits | 4.8 ± 10.7 | 5.3 ± 4.6 | 185.5 ± 195.4 | 6.8 ± 3.0 | 59.1 ± 41.1 | 185.9 ± 23.4 |
| Vegetables | 17.5 ± 26.6 | 45.1 ± 25.6 | 386.5 ± 330.8 | 12.3 ± 6.8 | 295.2 ± 248.4 | 418.1 ± 44.34 |
| UFG | 35.0 ± 40.8 | 108.8 ± 73.9 | 754.2 ± 581.8 | 22.7 ± 10.8 | 143.3 ± 76.3 | 912.5 ± 189.1 |
| SSB | 25.5 ± 28.4 | 63.7 ± 42.1 | 353.9 ± 176.0 | 17.1 ± 7.7 | 60.4 ± 30.1 | 522.7 ± 133.0 |
| UPF | 31.9 ± 35.2 | 76.9 ± 46.7 | 544.6 ± 373.9 | 19.5 ± 8.6 | 83.6 ± 34.7 | 691.5 ± 178.8 |
| UXFG | 22.3 ± 24.8 | 63.7 ± 42.1 | 514.4 ± 435.8 | 16.7 ± 7.5 | 56.4 ± 16.1 | 556.9 ± 128.7 |

Notes: HFG: healthy food group. UFG: unhealthy food group. UXFG: unhealthy in excessive amounts food group. SSB: sugar-sweetened beverages using the definition by the World Health Organization (WHO). UPF: ultra-processed foods. Values are mean (SD).

**Supplementary Table 12. Kernel density estimates (KDE) of food outlets around adolescents’ home and school by geographical location using different radii**

|  | **Within 200-meter radius** | | | **Within 400-meter radius** | | |
| --- | --- | --- | --- | --- | --- | --- |
|  | **Rural** | **Peri-urban** | **Urban** | **Rural** | **Peri-urban** | **Urban** |
|  | **Mean** ± **SD** | **Mean** ± **SD** | **Mean** ± **SD** | **Mean** ± **SD** | **Mean** ± **SD** | **Mean** ± **SD** |
|  |  |  |  |  |  |  |
| **Home food environment** | n = 1,067 | n = 933 | n = 663 | n = 1,067 | n = 933 | n = 663 |
| **KDE all outlets** | 1.8 ± 4.5 | 10.4 ± 28.4 | 68.8 ± 41.0 | 5.0 ± 10.5 | 36.9 ± 69.7 | 257.3 ± 61.8 |
| **KDE of outlets with food group** |  |  |  |  |  |  |
| HFG | 1.4 ± 3.5 | 8.1 ± 23.5 | 48.8 ± 31.6 | 3.9 ± 8.1 | 28.4 ± 55.9 | 180.6 ± 44.6 |
| Fruits | 0.4 ± 1.3 | 1.3 ± 4.3 | 11.6 ± 10.7 | 1.2 ± 2.9 | 5.2 ± 10.9 | 41.6 ± 16.3 |
| Vegetables | 0.8 ± 1.8 | 5.3 ± 19.0 | 22.2 ± 13.9 | 2.1 ± 4.1 | 17.9 ± 41.8 | 82.4 ± 20.9 |
| UFG | 1.4 ± 3.1 | 5.1 ± 8.8 | 48.9 ± 23.2 | 3.7 ± 6.8 | 17.6 ± 22.4 | 187.6 ± 43.1 |
| SSB | 1.0 ± 2.0 | 2.4 ± 2.9 | 24.6 ± 11.7 | 2.8 ± 4.0 | 7.4 ± 7.0 | 99.6 ± 27.8 |
| UPF | 1.2 ± 2.6 | 3.4 ± 4.7 | 35.2 ± 15.9 | 3.2 ± 5.5 | 11.4 ± 11.9 | 138.2 ± 34.2 |
| UXFG | 1.2 ± 2.7 | 3.0 ± 4.9 | 30.7 ± 15.9 | 3.2 ± 5.6 | 10.4 ± 12.1 | 114.9 ± 28.7 |
|  |  |  |  |  |  |  |
| **School food environment** | n = 5 | n = 3 | n = 4 | n = 5 | n = 3 | n = 4 |
| **KDE all outlets** | 2.2 ± 2.8 | 6.1 ± 5.4 | 60.9 ± 59.4 | 5.6 ± 4.6 | 34.6 ± 25.2 | 209.0 ± 107.0 |
| **KDE of outlets with food group** |  |  |  |  |  |  |
| HFG | 1.9 ± 2.5 | 3.8 ± 3.5 | 47.2 ± 45.9 | 4.3 ± 3.8 | 25.6 ± 19.1 | 149.0 ± 76.9 |
| Fruits | 0.3 ± 0.8 | 0.4 ± 0.4 | 12.3 ± 13.3 | 1.0 ± 1.0 | 3.5 ± 3.1 | 32.3 ± 19.0 |
| Vegetables | 1.0 ± 1.6 | 2.1 ± 2.0 | 22.3 ± 19.6 | 2.4 ± 2.5 | 18.5 ± 14.1 | 73.2 ± 36.4 |
| UFG | 2.0 ± 2.4 | 6.1 ± 5.5 | 40.3 ± 35.8 | 5.0 ± 4.0 | 21.6 ± 13.8 | 154.9 ± 73.5 |
| SSB | 1.6 ± 1.8 | 3.5 ± 3.1 | 17.6 ± 11.3 | 3.8 ± 3.0 | 12.3 ± 7.1 | 83.5 ± 28.4 |
| UPF | 1.9 ± 2.2 | 4.2 ± 3.7 | 30.3 ± 24.4 | 4.5 ± 3.6 | 15.8 ± 8.9 | 117.7 ± 50.1 |
| UXFG | 1.2 ± 1.3 | 3.0 ± 2.8 | 29.8 ± 27.0 | 3.2 ± 2.2 | 11.0 ± 5.4 | 98.7 ± 52.8 |

Notes: HFG: healthy food group. UFG: unhealthy food group. UXFG: unhealthy in excessive amounts food group. SSB: sugar-sweetened beverages using the definition by the World Health Organization (WHO). UPF: ultra-processed foods. Values are mean (SD).
